# 

**Published:** 1899-09-09

**Authors:** 


### NOTICE TO CORRESPONDENTS.

All MS., Letters, Books for Review, and other matters intended for the Editor should be addressed The Editor, "The Hospital," The Hospital Building, 28 & 29, Southampton Street, Strand, W.C.

The Editor cannot undertake to return rejected MS., even when accompanied by stamped directed envelope.

**Notice to Advertisers and Subscribers.**

All Advertisements, Orders for Copies of The Hospital, and Business Communications should be addressed to **THE MANAGER (Not to the Editor),** The Hospital, The Hospital Building, 28 & 29, Southampton Street, Strand, London, W.C.

The Cost of Subscription, post free, is as follows:—Per week, 2½d.; per quarter, 2s. 8d.; per half-year, 5s. 3d.; per annum, 10s. 6d. Foreign:—Per annum, 15s. 2d., payable, in all cases, in advance.

**NOTICE.—Vol. XXV. of "The Hospital" (October, 1898, to March, 1899), in handsome cloth binding, is now on sale at the Office of this Journal, price 7s. 6d. Cases for binding the Numbers contained in this Volume 1s. 6d. Index to Vol. XXV., 2½d., post free.**

**The Monthly Part for September is now ready. Price 6d., by post 8d.**

### Scraps and Gleanings.

During the past year 622 in-patients and 2,740 out-patients have been treated at the North Devon Infirmary.

The Commissioners have intimated their intention to supply water to the Dundee Victoria Hospital free of charge.

During the past year 307 in-patients, 2,713 out-patients, and 237 casualties were treated at Noble's Isle of Man Hospital.

The recent three days' bazaar held at Clacton in aid of the Clacton and District Cottage Hospital resulted in a sum of £903 being realised.

The cost of the enlargements to the Stephen Cottage Hospital, Dufftown, which is being generously defrayed by Lord Mount Stephen, will be £700.

The amount distributed by the Newcastle Sunday Fund since 1872, when the movement was got into thorough working order, has reached a total of £106,955.

At a recent meeting of the Forfar District Committee of Forfar City Council it was decided that the expenditure on the erection of the Dundee and Forfar Epidemic Hospital, exclusive of furnishings and the wall, should be £7,500.

Hospital Saturday in Hartlepool is aptly termed "Buttonhole Saturday," pedestrians, cyclists, and others being buttonholed in order that they may purchase buttonholes of flowers, all the profit from which sales goes to the hospital.

At a meeting of the Paignton Cottage Hospital the rules brought forward relating to the establishment of a provident dispensary were all passed. The expected cost to the hospital of the establishment of this dispensary will be about £25 to £30 per annum.

In consequence of the representations of the Organising Committee the Smallwood Hospital, Redditch, the medical men connected therewith have adopted the suggestion that all cases of illness, accident or otherwise, admitted to the hospital shall be treated absolutely free henceforward.

It has been decided, owing to the growth of the work done by the Seafield Private Hospital for Women, Dundee, to extend the existing accommodation by renting the house adjoining the present quarters. The proposed addition and alteration will give the hospital six or seven new beds at a cost of £300.

Owing to the time condition upon which the temporary isolation hospital provided by Miss Bromhead, of Lincoln, expiring in 18 months, the Lincoln Town Council have applied to the Local Government Board for sanction to borrow £1,167 for the purchase of land on which to erect an infectious diseases hospital.

At a meeting of the Caistor Rural Council it was resolved that the Caistor and Market Rasen authorities be asked to grant the necessary powers for proceeding without delay with the erection on the site already purchased of an isolation hospital, consisting of two wards to accommodate six persons in each ward.

At the recent quarterly Court of Governors of Addenbrooke's Hospital the question of the want of funds was again brought under notice. At that meeting it was announced that the deficiency was £1,000, whilst at the corresponding period of last year the deficiency was only £594. It has been decided to allow matters to slide until Michaelmas, on the chance of something turning up to obviate the necessity of selling out stock.

At a meeting convened by the Mayor of Southampton at the request of some 80 ratepayers the resolution was unanimously carried: "That, feeling the weight of the excessive rates of the town, those present are of opinion that the question of building a new workhouse infirmary, at a cost of £75,000, is altogether unnecessary, and this meeting calls upon the Guardians to abandon the proposed extravagant scheme (at Shirley Warren)."

The subscribers to the Diamond Jubilee Fund at Karachi have expressed their willingness to endow the Karachi Civil Hospital with sufficient funds to defray two-thirds of the salaries of a competent nurse, a Brahmin cook, and a high caste Hindoo ward boy, provided Government will grand the remaining one-third. They also agree to contribute on the same condition and same proportion the cost of erecting a bungalow for the nurse, a kitchen and quarters for the Brahmin cook and the ward boy, the cost of the buildings being roughly estimated at Rs.12,000 to Rs.15,000. The Diamond Jubilee Fund amounts to Rs.36,000.

The report of the Special Committee of the Glasgow Parish Council on poorhouse and hospital accommodation has been adopted, and a committee has been appointed to take steps for the purchase of the necessary land for the erection of a large general hospital to contain 1,200 patients, and two district hospitals, to accommodate 200 patients each, for the treatment of acute cases in the east and west ends of Glasgow respectively. The large general hospital will include a sanatorium for cases of consumption, wards for epileptics, and for the homeless poor persons discharged from the asylums. The report also advises the sale of the City Poorhouse and grounds in Parliament Road.

Contrary to some statements which have appeared in the press, the ear and throat department of the Eye Hospital, Southamption, has not been closed through want of funds, but because Dr. Powell, who has had charge of this department for the last nine years, resigned on account of pressure of other work, and the committee thereupon decided to utilise the whole space and strength at their disposal in the treatment of eye cases alone, which are so numerous as to render such a course advisable. Even with this additional space at command the committee consider that the present hospital accommodation is insufficient even for the work of the eye department alone, and they appeal for increased support to allow some more beds to be opened for eye cases and an additional out-patients' day to be arranged.

The Stanley Hospital, Liverpool, have received a special grant of £200 from the Liverpool Hospital Saturday and Sunday Fund, in view of the recent heavy expense to which the hospital has been put.

The formal opening by Lady Brassey of the Queen Victoria Hospital for Women and Children, Melbourne, took place in July last. This hospital is the Victorian women's Jubilee memorial of the Queen's reign, and the funds for it (£3,400) were collected in shillings from Victorian women of all classes.

The average number of in-patients at the Royal Cornwall Infirmary, Truro, last year was 31, the cost per bed being £61 6s. 3d.

As a memorial of the good work done for the Newcastle-on-Tyne Lying-in Hospital by the late Dr. Cargill Nesham, an appeal is being made to the public of Newcastle and adjoining counties for funds to enable the governors to place the lying-in hospital in a sound financial position to (1) build a new hospital, or (2) make such alterations and additions to the present hospital as the fund will permit.

### THE STUDENT OF MEDICINE.

### APPOINTMENTS.

E. W. Adams, M.B.Lond., appointed House Physician to the Northern Hospital, Liverpool. F. H. de G. Best, M.R.C.S., L.R.C.P.Lond., appointed Public Vaccinator to the Cheshunt District of the Edmonton Union. P. Best, M.R.C.S., L.R.C.P., appointed Medical Officer for the Third District of the Penzance Union. L. Black, M.B., B.C.Camb., appointed Medical Officer of Health to the Braintree Rural District Council. J. D. Doherty, M.D.Edin., appointed House Surgeon to the Northern Hospital, Liverpool. H. Hallam, M.R.C S., appointed Assistant Medical Officer to the Workhouse of the Eccleshall Union. H. W. Haydon, M.R.C.S.Eng., L.R.C.P., appointed Medical Officer of Health for the Wadebridge Urban District. W. Johnston, L R.C.P., M.R.C.S., L.S.D.Eng., appointed Honorary Visiting Dental Surgeon to the Somerset Hospital, Cape Town. W. M. Keal, L.R.C.P., L.R.C.S.Edin., appointed Medical Officer of the Workhouse and the Oakham District of the Oakham Union. A. Kinsey-Morgan, M.R.C.S., L.R.C.P., appointed House Surgeon to the Royal Victoria Hospital, Bournemouth. Miss B. Knowles, M.B., B.S.Lond., appointed Second Assistant Medical Officer to the Waterloo Road Workhouse, Bethnal Green. J. H. Meacher, M R.C.S., L.R.C P., appointed Medical Officer for the Second District of the Bodmin Union. S. C. Moore, M.B., Ch.B.Vict., appointed Assistant House Surgeon to the Northern Hospital, Liverpool. J. H. Muncaster, B.A.Camb., M.B.Lond., appointed Medical Officer for the North and Northowan Wards of the Halifax Union. F. O'Mara, L.R.C.P., L.R.C.S.Irel., appointed Resident Medical Superintendent of the Ennis District Lunatic Asylum. C. J. Utley, M.B.Lond., appointed Assistant Medical Officer to the Salford Union Infirmary. H. J. C. Wallace, L.R.C S.I., L.R.C.P.I., appointed Assistant Surgeon and Registrar to the Down County Infirmary. T. A. Buck, M.B.Lond., appointed Admiralty Surgeon and Agent at Ryde, Fishbourne, and Seaview. C. J Caddick, M.B., C.M., appointed House Surgeon to the Walsall and District Hospital. E. E. Crowther, L.R.C.P.Edin., L.R.C S.Edin., L.F.P.S.Glasg., appointed Medical Officer for the Luddenden District of the Halifax Union. E. W. Du Buisson, L.R.C.P.Lond., M.R C.S.Eng., appointed Medical Officer to the Dewchurch District of the Hereford Union. H. F. E. Harrison, L.R C.P.Lond., M.R C.S.Lond., appointed Medical Officer to the Third District of the Parish of Hammersmith. A. G. Ince, M.R C.S., L.R.C.P, appointed Medical Officer for the Fourth District of the Blean Union. W. E. Jones, M.R.C.S, L R.C.P.Lond, appointed Senior House Surgeon to the Halifax Infirmary. J. M. Longford, L.R.C.P.I. & L.M., L.R.C.S.I. & L M., appointed Assistant Medical Officer of the Workhouse of the Halifax Union. G. D. Maynard, M.R.C.S.Eng., L.R C.P.Lond., appointed Junior Out-patient Surgical Officer to the Royal London Ophthalmic Hospital, City Road. T. E. Nuttall, M.B., C.M.Edin, appointed Medical Officer for the First Accrington District of the Haslingden Union. J. Reid, M.D.Glasg., M.B., C.M., appointed Medical Officer of the First District of the Parish of Hammersmith. J. B. Stewart, M.A.Glasg., M.B., C.M., appointed Medical Officer of the Workhouse of the Haslingden Union. R. Sturgis White, M.B., C.M.Edin., appointed Medical Officer for the Cradley District of the Stourbridge Union. R. Wood, M.D.St.And., L.R.C.P.Lond., L.R.C.P.Edin., appointed Medical Officer for Bromsgrove.

